# New nomenclature combinations in the green alder species complex (Betulaceae)

**DOI:** 10.3897/phytokeys.56.5225

**Published:** 2015-08-14

**Authors:** Joyce Chery

**Affiliations:** 1 Department of Integrative Biology, University of California, Berkeley, California 94720

**Keywords:** Green alders, *Alnus viridis*, *Alnus alnobetula*, Betulaceae

## Abstract

The name *Alnus viridis* (Chaix) DC., based on *Betula viridis* Chaix (1785), has traditionally been attributed to green alders although it is based on a later basionym. *Alnus alnobetula* (Ehrh.) K. Koch based on *Betula alnobetula* Ehrh. (1783) is the correct name for green alders. In light of the increasing use and recognition of the name *Alnus alnobetula* (Ehrh.) K. Koch in the literature. I herein propose new nomenclatural combinations to account for the Japanese and Chinese subspecies respectively: Alnus alnobetula subsp. maximowiczii (Callier ex C.K. Schneid.) J. Chery and Alnus alnobetula subsp. mandschurica (Callier ex C.K. Schneid.) J. Chery. Recent phylogenetic analyses place these two taxa in the green alder species complex, suggesting that they should be treated as infraspecific taxa under the polymorphic *Alnus alnobetula*.

## Introduction

Characteristic to the genus, *Alnus alnobetula* (Ehrh.) K. Koch is an anemophilous shrub with carpellate catkins that develop into woody strobili. It has a circumpolar distribution with subspecies in Europe (Greuter and Raab-Straube 2011, Flora Europea [http://rbg-web2.rbge.org.uk/FE/fe.html – accessed 22.07.2015], North America (Furlow 1979, Furlow 1990, Fl. North. Amer. North of Mexico Editorial Committee 1997), and Asia (Löve 1968, Li and Skvortsov 1999, Ohba 2006). A phylogeny using nuclear ribosomal DNA ITS sequences generated a polytomy containing five taxa within the green alder species complex due to low sequence divergence among the individuals (Chen and Li 2004). Ren et al. (2010) found the green alder species complex to be a monophyletic clade with the unique character state of a thymine at position 192 of the ITS region. Banaev and Adel’shin (2009) also found close affinity of green alder species using molecular data.

The name *Alnus viridis* (Chaix) DC. has long been attributed to green alders; however a closer look at the literature reveals the name *Alnus alnobetula* (Ehrh.) K. Koch has priority (Pouzar 1982, Holub 1986). Appropriate nomenclatural combinations have recently been published for Alnus alnobetula subsp. crispa (Aiton) Raus, Alnus alnobetula subsp. sinuata (Aiton) Raus (Greuter and Raab-Straube 2011), and Alnus alnobetula subsp. suaveolens (Req.) Lambinon & Kerguélen (Lambinon and Kerguélen 1988). Subspecies names for the Japanese green alder and Chinese green alder are assigned here.

## Nomenclature history

The confusion lies in the appropriate basionym of this taxon. The name *Betula viridis* Chaix dates from 1785 (unable to access original text; revisited in Perret and Burdet 1981). No type specimen was designated. Two years earlier, *Betula alnobetula* Ehrh. was published by Ehrhart (in Gartenkalender 1783) describing a shrub in which “the homeland is unknown to me” (translated from German). In Ehrhart (1788), he republished his work where the name *Betula alnobetula* Ehrh. reappeared.

As *Betula* species were transferred to *Alnus*, authors were evidently unaware of the original 1783 publication of the name *Betula alnobetula* Ehrh., so *Betula viridis* Chaix was thought to be the older name and was taken to be the basionym for green alders. *Alnus alnobetula* Ehrh. has consistently been associated with the 1788 reproduced work and thus listed as a later synonym of *Alnus viridis* (Chaix) DC.

Major databases such as plantlist.org [accessed 22.07.2015], list the name *Alnus viridis* (Chaix) DC. as a synonym of *Alnus alnobetula* (Ehrh.) K. Koch. Other databases seem to be waiting for formal action to account for all subspecies names. For example, USDA, Germplasm Resources Information Network (GRIN 2015) [http://www.ars-grin.gov/cgi-bin/npgs/html/taxon.pl?2483], states: “the name *Alnus alnobetula* (Ehrh.) K. Koch, based on *Betula alnobetula* Ehrh. (1783) has priority over *Alnus viridis* (Chaix) DC., based on *Betula viridis* Chaix (1786); nevertheless, *Alnus viridis* is retained here until all infraspecific taxa are accounted for under *Alnus alnobetula*”. Other major databases have incomplete citation list for synonyms such as Fl. North Amer. North of Mexico Editorial Committee [http://www.efloras.org/flora_page.aspx?flora_id=1 – accessed 22.07.2015]. Flora Europea [http://rbg-web2.rbge.org.uk/FE/fe.html – accessed 22.07.2015] omits citations for green alder names.

## Conclusions

The close relatedness of the green alder species complex members is supported by recent phylogenetic anaylses. The use of a single nrDNA marker, ITS, generated a weakly supported clade of *Alnus mandshurica*, *Alnus firma*, *Alnus pendula* and *Alnus sieboldiana* embedded within a greater polytomy that includes all other green alders (see strict consensus parsimony tree by Chen and Li 2004). In more recent phylogenetic analysis, *Alnus maximowiczii* and *Alnus mandshurica* always form a monophyletic clade with the rest of the green alders (Ren et al. 2010, Banaev and Adel’shin 2009). Given this evidence, it is appropriate to change the rank of these taxa to subspecies of the green alders. The proposed nomenclature changes utilize the correct species epithet and recognize their phylogenetic placement as lineages of a polymorphic *Alnus alnobetula*.

Infraspecific rankings of plants, specifically subspecies and variety, have been used rather interchangeably (Hamilton and Reichard 1992). The green alder species complex has historically been separated into subspecies due to geographic and morphological distinctiveness. I here agree with this subspecies concept and propose two new nomenclatural combinations to account for the Japanese and Chinese green alder subspecies. This change provides the proper nomenclature for future taxonomic and phylogenetic studies in the green alder species complex.

### Alnus alnobetula subsp. maximowiczii

Taxon classificationPlantaeFagalesBetulaceae

(Callier ex C.K. Schneid.) Chery comb. n.

urn:lsid:ipni.org:names:77149153-1

Alnus maximowiczii Callier ex C.K. Schneid., Illustr. Handb. Laubholzk. 1: 122. 1904: typified by the plate accompanying the protologue (Basionym).
Alnus crispa subsp. maximowiczii (Callier ex C.K. Schneid.) Hultén, Acta Univ. Lund. Avd. 2. 40(1): 590. 1944.
Alnaster maximowiczii (Callier) Czerep., Bot. Mater. Gerb. Bot. Inst. Komarova Akad. Nauk. S.S.S.R. 17: 97. 1955.
Alnaster crispus subsp. maximowiczii (Callier ex C.K. Schneid.) Murai, Bull. Gov. Forest Exp.Sta.154: 62. 1963.
Duschekia maximowiczii (Callier ex C.K. Schneid.) Pouzar, Preslia 36: 339. 1964.
Alnaster maximowiczii (Callier) Czerep., Fl. Arct. URSS Fasc. 5, 133 in obs. 1966.
Alnus viridis subsp. maximoviczii (Callier ex C.K. Schneid.) D. Löve, Taxon 17: 89. 1968.
Alnus viridis subsp. maximowiczii (Callier ex C.K. Schneid.) H. Ohba, Fl. Japan 2a: 27. 2006.

#### Distribution.

Temperate Asia: Russian Federation - Khabarovsk, Kurile Islands, Primorye, Sakhalin; Japan - Hokkaido, Honshu; Korea

**Figure 1.**
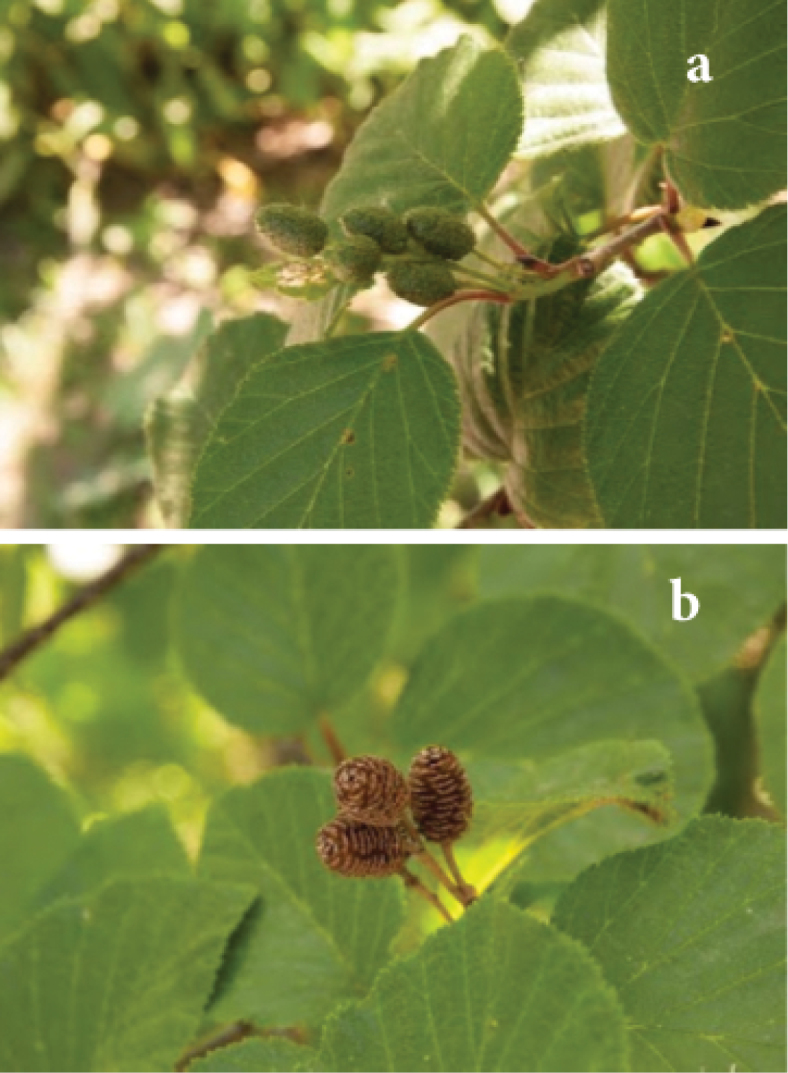
Alnus alnobetula subsp. maximowiczii – images (taken by Jordan Wood) from Arnold Arboretum 1462-77*E **a**) developing infructescenes; **b**) old infructescences.

### Alnus alnobetula subsp. mandschurica

Taxon classificationPlantaeFagalesBetulaceae

(Callier ex C.K. Schneid.) Chery comb. n.

urn:lsid:ipni.org:names:77149155-1

Alnus fruticosa var. mandschurica Callier ex C.K. Schneid., Illustr. Handb. Laubholzk. 1:121. 1904: Lectotype: Nadelholzzone des Tschangpei-schan, immer vereinzelt, 1600–1800 m (Fenze 262); designated by Hand.-Mazz., not seen) (Basionym).
Alnus fruticosa var. mandschurica Callier ex Kom., Acta Hort. Petr. 22: 59. 1903.
Alnus fruticosa var. mandschurica f. normalis Callier, Fedde, Rep. Spec. Nov. 10: 227. 1911.
Alnus fruticosa var. mandschurica f. grandifolia Callier, Fedde, Rep. Spec. Nov. 10: 227.1911.
Alnus mandschurica (Callier ex C.K. Schneid.) Hand.-Mazz., Oesterr. Bot. Z. 81: 306–307.1932.
Alnus crispa (Aiton) Pursh subsp. mandshurica (Callier) Hara, J. Fac. Sci. Univ. Tokyo III, -6, (2): 32. 1952.
Alnus mandschurica var. pubescens Baranov, in T. N. Liou, Illustrated Flora of Ligneous plants of N. E. China 206, t. 75, fig. 112, t. 76, figs 1–4. 1955.
Duschekia mandschurica (Callier ex C.K. Schneid.) Pouzar, Preslia 36(4): 339. 1964.
Alnaster crispa (Aiton) ssp. mandshurica (Callier) Murai, Bull. Gov. For. Expt. Sta. Jap. 171: 34. 1964.

#### Distribution.

Russian Federation: Khabarovsk, Primorye; China: Heilongjiang, Jilin, Liaoning, Nei Monggol; Korea

## Supplementary Material

XML Treatment for Alnus alnobetula subsp. maximowiczii

XML Treatment for Alnus alnobetula subsp. mandschurica

## References

[B1] Banaev EV Adel’shin RV (2009) Structure of *Alnus fruticosa* Rupr. s.l. and its relationships with other taxa of subgenus Alnobetula (Ehrhart) Peterman. Contemporary Problems of Ecology 2(6): 601–610. doi: 10.1134/S1995425509060186

[B2] Chen Z Li J (2004) Phylogenetics and biogeography of *Alnus* (Betulaceae) inferred from sequences of nuclear ribosomal DNA ITS region. International Journal of Plant Sciences 165(2): 325–335. doi: 1086/382795

[B3] Ehrhart JF (1783) Bestimmung einiger Bäume und Sträucher aus unseren Lustgebüschen.

[B4] Gartenkalender 2: 193. http://www.ub.uni-bielefeld.de/cgi-bin/neubutton.cgi?pfad=/diglib/aufkl/gartenkal/095831&seite=00000248.TIF

[B5] Ehrhart JF (1788) Beiträge zur Naturkunde, und den damit verwandten wissenschaften, besonders der botanik, chemie, haus- und landwirthschaft, arzneigelahrtheit und apothekerkunst vol (Bd 2) Hannover & Osnabrück, 67–72. doi: 10.5962/bhl.title.44806

[B6] Hamilton CW Reichard SH (1992) Current practice in the use of subspecies, variety, and forma in the classification of wild plants. Taxon 41(3): 485–498. doi: 10.2307/1222819

[B7] Furlow JJ (1979) The Systematics of the American Species of *Alnus* (Betulaceae). Rhodora 81(825): 1–121. http://biostor.org/reference/137992

[B8] Furlow JJ (1990) The genera of Betulaceae in the southeastern United States. Journal of the Arnold Arboretum 71: 1–67. http://biostor.org/reference/61852

[B9] GRIN (2015) Germplasm Resources Information Network. http://www.ars-grin.gov/cgi-bin/npgs/html/index.pl [accessed 22.07.2015]

[B10] Greuter W Von Raav-Straube E (2011) Euro+Med Notulae, 5. Willdenowia 41(1): 129–138. doi: 10.3372/wi.41.41117

[B11] Holub J (1986) Comments on the “Med-Checklist 1”. Preslia 58: 289–306.

[B12] Ohba H (2006) *Alnus*. Flora of Japan Vol. 2a, Angiospermae, Dicotyledoneae, Archichlamydeae(a). Kodansha, Tokyo, 26–31.

[B13] Koch K (1872) Dendrologie. Bäume, Sträucher und Halbsträucher, welche in Mittel- und Nord Europa im Freien kultivirt werden (Vol 2). F. Enke, Erlangen, 623–638. doi: 10.5962/bhl.title.20459

[B14] de Lamarck J Candolle A (1805) Flore française: ou descriptions succinctes de toutes les plantes qui croissent naturellement en France, disposées selon une novelle méthode d’analyse, et précédées par un exposé des principes élémentaires de la botanique (Vol 3). H. Agasse, Paris, 304 pp http://hdl.handle.net/2027/hvd.32044107276479?urlappend=%3Bseq=354

[B15] Lambinon J Kerguélen L (1988) Trois combinaisons nomenclatures nouvelles relatives à la flore corse. Candollea 43: 405–406.

[B16] Li P Skvortsov AK (1999) Betulaceae. Flora of China Vol 4: Cycadaceae through Fagaceae. Science Press, Beijing, and Missouri Botanical Garden Press, St. Louis, 286–313. http://www.efloras.org/florataxon.aspx?flora_id=2&taxon_id=10101

[B17] Löve D (1968) Nomenclatural Notes on Mt. Washington Plants. Taxon 17(1): 89. doi: 10.2307/1216168

[B18] Pandora Taxonomic Database System (digital version of the Flora Europaea). Royal Botanic Garden Edinburgh http://rbg-web2.rbge.org.uk/FE/fe.html [accessed 22.07.2015]

[B19] Perret P Burdet HM (1981) 2. Les “Plantae Vapincenses” de Dominique Chaix et les travaux floristiques de Dominique Villars en Dauphiné. In: Burdet HM (Ed.) Med-Checklist Notulae Bibliographicae, 1 et 2 Candollea 36: 400–408.

[B20] Pouzar Z (1982) Otázka správného jména pro olši zelenou. Journal of the National Museum (Prague), Natural History, 151: 20.

[B21] Ren B Xiag X Chen Z (2010) Species identification of *Alnus* (Betulaceae) using nrDNA and cpDNA genetic markers. Molecular Ecology Resources 10(4): 594–605. doi: 10.1111/j.1755-0998.2009.02815.x 21565064 10.1111/j.1755-0998.2009.02815.x

